# Benzyl *N*′-(1-methyl-1*H*-indol-2-ylmethyl­ene)hydrazinecarbodithio­ate

**DOI:** 10.1107/S1600536808038579

**Published:** 2008-11-26

**Authors:** Hamid Khaledi, Hapipah Mohd Ali, Seik Weng Ng

**Affiliations:** a Department of Chemistry, University of Malaya, 50603 Kuala Lumpur, Malaysia

## Abstract

In the title compound, C_18_H_17_N_3_S_2_, the dihedral angle between the planes of the aromatic ring systems is 83.63 (16)°. In the crystal structure, inversion dimers occur, linked by pairs of N—H⋯S hydrogen bonds.

## Related literature

For the crystal structures of the benzyl esters of hydrazinecarbodithioic acids, see: Ali *et al.* (2004 ▶); Chan *et al.* (2003 ▶); Fun *et al.* (1995 ▶); How *et al.* (2007 ▶); Khoo *et al.* (2005 ▶); Qiu & Luo (2007 ▶); Roy *et al.* (2007 ▶); Tarafder *et al.* (2002 ▶); Xu *et al.* (1991 ▶, 2002 ▶); Zhang *et al.* (2004 ▶).
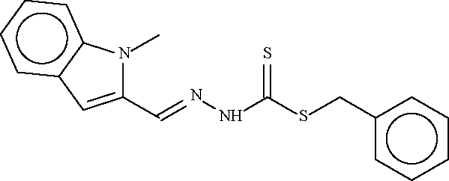


## Experimental

### 

#### Crystal data

C_18_H_17_N_3_S_2_
*M* *_r_* = 339.47
Triclinic, 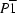

*a* = 5.1203 (1) Å
*b* = 11.9377 (3) Å
*c* = 14.1936 (4) Å
α = 108.203 (2)°
β = 90.230 (2)°
γ = 96.191 (2)°
*V* = 818.70 (3) Å^3^
*Z* = 2
Mo *K*α radiation
μ = 0.33 mm^−1^
*T* = 100 (2) K
0.40 × 0.08 × 0.04 mm

#### Data collection

Bruker SMART APEX diffractometer
Absorption correction: multi-scan (*SADABS*; Sheldrick, 1996 ▶) *T*_min_ = 0.880, *T*_max_ = 0.987
7605 measured reflections
3732 independent reflections
2653 reflections with *I* > 2σ(*I*)
*R*_int_ = 0.047

#### Refinement

*R*[*F* ^2^ > 2σ(*F* ^2^)] = 0.075
*wR*(*F* ^2^) = 0.225
*S* = 1.13
3732 reflections
209 parameters
H-atom parameters constrained
Δρ_max_ = 1.56 e Å^−3^
Δρ_min_ = −0.59 e Å^−3^

### 

Data collection: *APEX2* (Bruker, 2007 ▶); cell refinement: *SAINT* (Bruker, 2007 ▶); data reduction: *SAINT*; program(s) used to solve structure: *SHELXS97* (Sheldrick, 2008 ▶); program(s) used to refine structure: *SHELXL97* (Sheldrick, 2008 ▶); molecular graphics: *X-SEED* (Barbour, 2001 ▶); software used to prepare material for publication: *pubCIF* (Westrip, 2008 ▶).

## Supplementary Material

Crystal structure: contains datablocks global, I. DOI: 10.1107/S1600536808038579/hb2848sup1.cif

Structure factors: contains datablocks I. DOI: 10.1107/S1600536808038579/hb2848Isup2.hkl

Additional supplementary materials:  crystallographic information; 3D view; checkCIF report

## Figures and Tables

**Table 1:** Hydrogen-bond geometry (Å, °)

*D*—H⋯*A*	*D*—H	H⋯*A*	*D*⋯*A*	*D*—H⋯*A*
N1—H1⋯S2^i^	0.88	2.49	3.336 (3)	161

Symmetry code: (i) 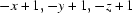.

**Fig. 1.**
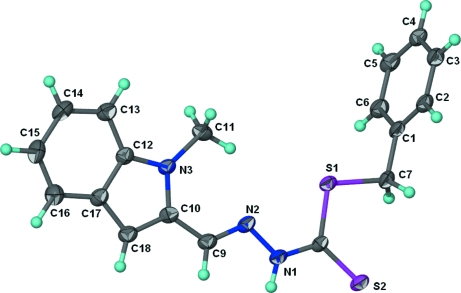
The molecular structue of (I) at the 70% probability level. Hydrogen atoms are drawn as spheres of arbitrary radius.

## supplementary crystallographic information

### Comment

For related structures, see: Ali *et al.* (2004); Chan *et al.* (2003); Fun *et al.* (1995); How *et al.* (2007); Khoo *et al.* (2005); Qiu & Luo (2007); Roy *et al.* (2007); Tarafder *et al.* (2002); Xu *et al.* (1991, 2002); Zhang *et al.* (2004).

### Experimental

1-Methylindole-2-carbaldehyde (0.40 g, 2.5 mmol) and *S*-benzyldithiocarbazate (0.50 g, 2.5 mmol) were heated in ethanol (40 ml) for 3 h. The solid that separated on cooling the solution was collected and recrystallized from ethanol and washed with cold ethanol and dried. Orange prisms of (I) were grown by slow evaporation of ethanol solution at room temperature.

### Refinement

Hydrogen atoms were placed at calculated positions (C–H = 0.95–0.99, N–H = 0.88Å) and refined as riding with *U*(H) = 1.2–1.5 times *U*_eq_(C,*N*).

The largest difference peak is close to S2.

### Crystal data

**Table:**

C_18_H_17_N_3_S_2_	*Z* = 2
*M**_r_* = 339.47	*F*_000_ = 356
Triclinic, *P*1	*D*_x_ = 1.377 Mg m^−^^3^
Hall symbol: -P 1	Mo *K*α radiation λ = 0.71073 Å
*a* = 5.1203 (1) Å	Cell parameters from 2060 reflections
*b* = 11.9377 (3) Å	θ = 2.6–28.1º
*c* = 14.1936 (4) Å	µ = 0.33 mm^−^^1^
α = 108.203 (2)º	*T* = 100 (2) K
β = 90.230 (2)º	Prism, orange
γ = 96.191 (2)º	0.40 × 0.08 × 0.04 mm
*V* = 818.70 (3) Å^3^	

### Data collection

**Table:**

Bruker SMART APEX diffractometer	3732 independent reflections
Radiation source: fine-focus sealed tube	2653 reflections with *I* > 2σ(*I*)
Monochromator: graphite	*R*_int_ = 0.047
*T* = 100(2) K	θ_max_ = 27.5º
ω scans	θ_min_ = 1.5º
Absorption correction: Multi-scan(SADABS; Sheldrick, 1996)	*h* = −6→6
*T*_min_ = 0.880, *T*_max_ = 0.987	*k* = −15→15
7605 measured reflections	*l* = −18→18

### Refinement

**Table:**

Refinement on *F*^2^	Secondary atom site location: difference Fourier map
Least-squares matrix: full	Hydrogen site location: inferred from neighbouring sites
*R*[*F*^2^ > 2σ(*F*^2^)] = 0.075	H-atom parameters constrained
*wR*(*F*^2^) = 0.225	*w* = 1/[σ^2^(*F*_o_^2^) + (0.1301*P*)^2^ + 0.2413*P*] where *P* = (*F*_o_^2^ + 2*F*_c_^2^)/3
*S* = 1.13	(Δ/σ)_max_ = 0.001
3732 reflections	Δρ_max_ = 1.56 e Å^−^^3^
209 parameters	Δρ_min_ = −0.59 e Å^−^^3^
Primary atom site location: structure-invariant direct methods	Extinction correction: none

### Fractional atomic coordinates and isotropic or equivalent isotropic displacement parameters (Å^2^)

**Table:**

	*x*	*y*	*z*	*U*_iso_*/*U*_eq_	
S1	0.58962 (17)	0.85808 (8)	0.65532 (7)	0.0205 (3)	
S2	0.72855 (18)	0.65218 (8)	0.48348 (7)	0.0221 (3)	
N1	0.3474 (6)	0.6436 (3)	0.6054 (2)	0.0201 (7)	
H1	0.3089	0.5687	0.5697	0.024*	
N2	0.2021 (6)	0.6924 (3)	0.6868 (2)	0.0195 (7)	
N3	−0.1582 (6)	0.7565 (3)	0.8569 (2)	0.0184 (7)	
C1	0.9083 (7)	1.0499 (3)	0.6464 (3)	0.0215 (8)	
C2	1.1083 (7)	1.1026 (3)	0.7164 (3)	0.0220 (8)	
H2	1.2181	1.0543	0.7367	0.026*	
C3	1.1524 (7)	1.2255 (4)	0.7581 (3)	0.0225 (8)	
H3	1.2914	1.2608	0.8062	0.027*	
C4	0.9918 (7)	1.2961 (3)	0.7288 (3)	0.0220 (8)	
H4	1.0218	1.3801	0.7563	0.026*	
C5	0.7885 (8)	1.2441 (4)	0.6596 (3)	0.0245 (8)	
H5	0.6772	1.2924	0.6402	0.029*	
C6	0.7460 (7)	1.1216 (4)	0.6183 (3)	0.0245 (8)	
H6	0.6058	1.0864	0.5707	0.029*	
C7	0.8657 (7)	0.9171 (3)	0.5981 (3)	0.0241 (8)	
H7A	1.0257	0.8814	0.6075	0.029*	
H7B	0.8278	0.8978	0.5260	0.029*	
C8	0.5454 (7)	0.7094 (3)	0.5804 (3)	0.0172 (7)	
C9	0.0151 (7)	0.6197 (3)	0.7016 (3)	0.0207 (8)	
H9	−0.0098	0.5417	0.6557	0.025*	
C10	−0.1597 (7)	0.6484 (3)	0.7829 (3)	0.0188 (8)	
C11	0.0120 (8)	0.8667 (3)	0.8688 (3)	0.0256 (9)	
H11A	0.1053	0.8922	0.9337	0.038*	
H11B	−0.0949	0.9284	0.8643	0.038*	
H11C	0.1397	0.8536	0.8163	0.038*	
C12	−0.3503 (7)	0.7444 (3)	0.9212 (3)	0.0194 (8)	
C13	−0.4243 (8)	0.8289 (4)	1.0078 (3)	0.0240 (8)	
H13	−0.3371	0.9075	1.0304	0.029*	
C14	−0.6278 (8)	0.7930 (4)	1.0584 (3)	0.0268 (9)	
H14	−0.6825	0.8482	1.1170	0.032*	
C15	−0.7569 (8)	0.6772 (4)	1.0260 (3)	0.0289 (9)	
H15	−0.8972	0.6556	1.0628	0.035*	
C16	−0.6840 (8)	0.5941 (4)	0.9418 (3)	0.0273 (9)	
H16	−0.7720	0.5157	0.9206	0.033*	
C17	−0.4769 (7)	0.6274 (3)	0.8878 (3)	0.0206 (8)	
C18	−0.3533 (7)	0.5682 (3)	0.8002 (3)	0.0218 (8)	
H18	−0.3958	0.4877	0.7605	0.026*	

### Atomic displacement parameters (Å^2^)

**Table:**

	*U*^11^	*U*^22^	*U*^33^	*U*^12^	*U*^13^	*U*^23^
S1	0.0212 (5)	0.0153 (5)	0.0253 (5)	0.0064 (3)	0.0069 (4)	0.0053 (4)
S2	0.0263 (5)	0.0180 (5)	0.0230 (5)	0.0078 (4)	0.0098 (4)	0.0060 (4)
N1	0.0221 (15)	0.0163 (15)	0.0211 (16)	0.0058 (12)	0.0055 (13)	0.0034 (12)
N2	0.0221 (15)	0.0181 (16)	0.0211 (16)	0.0090 (12)	0.0056 (13)	0.0081 (13)
N3	0.0220 (15)	0.0131 (15)	0.0230 (16)	0.0065 (12)	0.0057 (13)	0.0081 (12)
C1	0.0207 (18)	0.0182 (19)	0.027 (2)	0.0074 (14)	0.0118 (15)	0.0076 (15)
C2	0.0193 (18)	0.0203 (19)	0.030 (2)	0.0093 (14)	0.0061 (15)	0.0101 (16)
C3	0.0194 (18)	0.023 (2)	0.025 (2)	0.0051 (14)	0.0031 (15)	0.0075 (16)
C4	0.0253 (19)	0.0205 (19)	0.0228 (19)	0.0067 (15)	0.0096 (15)	0.0090 (15)
C5	0.029 (2)	0.023 (2)	0.028 (2)	0.0122 (16)	0.0087 (17)	0.0129 (16)
C6	0.0219 (19)	0.025 (2)	0.028 (2)	0.0079 (15)	0.0019 (16)	0.0074 (16)
C7	0.0189 (17)	0.021 (2)	0.033 (2)	0.0059 (14)	0.0093 (16)	0.0069 (16)
C8	0.0171 (16)	0.0173 (18)	0.0192 (17)	0.0078 (13)	0.0024 (14)	0.0069 (14)
C9	0.0236 (18)	0.0152 (18)	0.0247 (19)	0.0069 (14)	0.0022 (15)	0.0067 (15)
C10	0.0232 (18)	0.0152 (18)	0.0210 (18)	0.0064 (14)	0.0006 (15)	0.0086 (14)
C11	0.031 (2)	0.0130 (18)	0.033 (2)	0.0006 (15)	0.0061 (17)	0.0077 (16)
C12	0.0201 (17)	0.0182 (18)	0.0227 (19)	0.0066 (14)	0.0018 (15)	0.0089 (15)
C13	0.0257 (19)	0.022 (2)	0.025 (2)	0.0067 (15)	0.0031 (16)	0.0066 (16)
C14	0.028 (2)	0.031 (2)	0.023 (2)	0.0126 (16)	0.0058 (16)	0.0069 (17)
C15	0.0226 (19)	0.037 (2)	0.029 (2)	0.0038 (16)	0.0067 (16)	0.0126 (18)
C16	0.027 (2)	0.027 (2)	0.028 (2)	0.0010 (16)	0.0059 (17)	0.0098 (17)
C17	0.0214 (18)	0.0199 (19)	0.0227 (19)	0.0075 (14)	0.0029 (15)	0.0084 (15)
C18	0.0256 (19)	0.0168 (18)	0.0243 (19)	0.0055 (14)	0.0047 (15)	0.0073 (15)

### Geometric parameters (Å, °)

**Table:**

S1—C8	1.750 (4)	C6—H6	0.9500
S1—C7	1.818 (4)	C7—H7A	0.9900
S2—C8	1.673 (4)	C7—H7B	0.9900
N1—C8	1.332 (5)	C9—C10	1.442 (5)
N1—N2	1.382 (4)	C9—H9	0.9500
N1—H1	0.8800	C10—C18	1.380 (5)
N2—C9	1.284 (5)	C11—H11A	0.9800
N3—C12	1.371 (5)	C11—H11B	0.9800
N3—C10	1.386 (5)	C11—H11C	0.9800
N3—C11	1.460 (5)	C12—C17	1.409 (5)
C1—C2	1.377 (5)	C12—C13	1.409 (5)
C1—C6	1.395 (5)	C13—C14	1.374 (6)
C1—C7	1.509 (5)	C13—H13	0.9500
C2—C3	1.392 (5)	C14—C15	1.401 (6)
C2—H2	0.9500	C14—H14	0.9500
C3—C4	1.387 (5)	C15—C16	1.377 (6)
C3—H3	0.9500	C15—H15	0.9500
C4—C5	1.380 (6)	C16—C17	1.405 (6)
C4—H4	0.9500	C16—H16	0.9500
C5—C6	1.388 (6)	C17—C18	1.415 (5)
C5—H5	0.9500	C18—H18	0.9500
			
C8—S1—C7	101.41 (18)	S2—C8—S1	124.0 (2)
C8—N1—N2	120.4 (3)	N2—C9—C10	124.5 (3)
C8—N1—H1	119.8	N2—C9—H9	117.7
N2—N1—H1	119.8	C10—C9—H9	117.7
C9—N2—N1	114.0 (3)	C18—C10—N3	109.2 (3)
C12—N3—C10	107.9 (3)	C18—C10—C9	123.9 (3)
C12—N3—C11	123.7 (3)	N3—C10—C9	126.9 (3)
C10—N3—C11	128.4 (3)	N3—C11—H11A	109.5
C2—C1—C6	118.9 (3)	N3—C11—H11B	109.5
C2—C1—C7	121.4 (3)	H11A—C11—H11B	109.5
C6—C1—C7	119.7 (4)	N3—C11—H11C	109.5
C1—C2—C3	121.1 (3)	H11A—C11—H11C	109.5
C1—C2—H2	119.5	H11B—C11—H11C	109.5
C3—C2—H2	119.5	N3—C12—C17	108.9 (3)
C4—C3—C2	119.5 (4)	N3—C12—C13	129.3 (4)
C4—C3—H3	120.2	C17—C12—C13	121.8 (4)
C2—C3—H3	120.2	C14—C13—C12	117.2 (4)
C5—C4—C3	119.9 (4)	C14—C13—H13	121.4
C5—C4—H4	120.1	C12—C13—H13	121.4
C3—C4—H4	120.1	C13—C14—C15	121.8 (4)
C4—C5—C6	120.2 (4)	C13—C14—H14	119.1
C4—C5—H5	119.9	C15—C14—H14	119.1
C6—C5—H5	119.9	C16—C15—C14	121.2 (4)
C5—C6—C1	120.3 (4)	C16—C15—H15	119.4
C5—C6—H6	119.8	C14—C15—H15	119.4
C1—C6—H6	119.8	C15—C16—C17	118.8 (4)
C1—C7—S1	108.2 (3)	C15—C16—H16	120.6
C1—C7—H7A	110.0	C17—C16—H16	120.6
S1—C7—H7A	110.0	C16—C17—C12	119.3 (4)
C1—C7—H7B	110.0	C16—C17—C18	134.3 (4)
S1—C7—H7B	110.0	C12—C17—C18	106.4 (3)
H7A—C7—H7B	108.4	C10—C18—C17	107.5 (3)
N1—C8—S2	121.5 (3)	C10—C18—H18	126.2
N1—C8—S1	114.4 (3)	C17—C18—H18	126.2
			
C8—N1—N2—C9	179.1 (3)	N2—C9—C10—C18	−177.4 (4)
C6—C1—C2—C3	−0.9 (6)	N2—C9—C10—N3	0.5 (6)
C7—C1—C2—C3	177.7 (4)	C10—N3—C12—C17	−0.2 (4)
C1—C2—C3—C4	0.2 (6)	C11—N3—C12—C17	179.8 (3)
C2—C3—C4—C5	0.7 (6)	C10—N3—C12—C13	179.8 (4)
C3—C4—C5—C6	−0.8 (6)	C11—N3—C12—C13	−0.2 (6)
C4—C5—C6—C1	0.0 (6)	N3—C12—C13—C14	179.4 (4)
C2—C1—C6—C5	0.8 (6)	C17—C12—C13—C14	−0.6 (6)
C7—C1—C6—C5	−177.8 (4)	C12—C13—C14—C15	0.3 (6)
C2—C1—C7—S1	102.5 (4)	C13—C14—C15—C16	0.2 (7)
C6—C1—C7—S1	−78.9 (4)	C14—C15—C16—C17	−0.3 (6)
C8—S1—C7—C1	172.9 (3)	C15—C16—C17—C12	0.0 (6)
N2—N1—C8—S2	178.8 (2)	C15—C16—C17—C18	−179.5 (4)
N2—N1—C8—S1	−2.4 (4)	N3—C12—C17—C16	−179.5 (3)
C7—S1—C8—N1	−179.1 (3)	C13—C12—C17—C16	0.5 (6)
C7—S1—C8—S2	−0.3 (3)	N3—C12—C17—C18	0.1 (4)
N1—N2—C9—C10	178.4 (3)	C13—C12—C17—C18	−179.9 (3)
C12—N3—C10—C18	0.2 (4)	N3—C10—C18—C17	−0.2 (4)
C11—N3—C10—C18	−179.8 (4)	C9—C10—C18—C17	178.1 (3)
C12—N3—C10—C9	−177.9 (3)	C16—C17—C18—C10	179.5 (4)
C11—N3—C10—C9	2.0 (6)	C12—C17—C18—C10	0.0 (4)

### Hydrogen-bond geometry (Å, °)

**Table:**

*D*—H···*A*	*D*—H	H···*A*	*D*···*A*	*D*—H···*A*
N1—H1···S2^i^	0.88	2.49	3.336 (3)	161

Symmetry codes: (i) −*x*+1, −*y*+1, −*z*+1.
